# Health Disparities in COVID-19: Addressing the Role of Social Determinants of Health in Immune System Dysfunction to Turn the Tide

**DOI:** 10.3389/fpubh.2020.559312

**Published:** 2020-10-08

**Authors:** Yvonne Baumer, Nicole Farmer, Thomas A. Premeaux, Gwenyth R. Wallen, Tiffany M. Powell-Wiley

**Affiliations:** ^1^Social Determinants of Obesity and Cardiovascular Risk Laboratory, Cardiovascular Branch, Division of Intramural Research, National Heart, Lung, and Blood Institute, National Institutes of Health, Bethesda, MD, United States; ^2^National Institutes of Health, Clinical Center, Bethesda, MD, United States; ^3^Intramural Research Program, National Institute on Minority Health and Health Disparities, Bethesda, MD, United States

**Keywords:** health disparities, COVID-19, IL-6, immune system, psychoneuroimmunology, psychosocial stress

## Abstract

It is evident that health disparities exist during the COVID-19 pandemic, a pandemic caused by the novel coronavirus SARS-CoV-2. Underlying reasons for COVID-19 health disparities are multi-factorial. However, social determinants, including those regarding socioeconomic status, social inequalities, health behaviors, and stress, may have implications on these disparities. Exposure to one or more of these social determinants is associated with heightened inflammatory responses, particularly increases in the cytokine interleukin-6 (IL-6), as well as immune system dysfunction. Thus, an amplified effect during COVID-19 could occur, potentially resulting in vulnerable patients experiencing an intensified cytokine storm due to a hyperactive and dysfunctional immune response. Further understanding how social determinants play a mechanistic role in COVID-19 disparities could potentially help reduce health disparities overall and in future pandemics.

## Introduction

As of August 26th 2020, more than 24 million people have positively tested for SARS-CoV-2, the virus responsible for the COVID-19 pandemic and more than 820,000 globally have died in this current COVID-19 pandemic, which is affecting people with underlying conditions more severely than others (1). In the United States, racial/ethnic disparities in COVID-19-related severity, mortality and outcomes exist. Although not the majority of cases, there is a disproportionate number of COVID-19-related deaths among Americans who are of Hispanic or African descent (2–5). Additionally, COVID-19 disproportionally affects ethnic minority communities in countries outside of the U.S. (6, 7). COVID-19 disparities have also been reported in the UK (8, 9), while the majority of countries have yet to publish data on race/ethnic-related disparities. Unfortunately, prior epidemics have also highlighted existing health disparities and prior discussions have emphasized the importance of incorporating anticipated health disparity outcomes into pandemic planning (10–13).

The underlying reasons for racial/ethnic health disparities are almost certainly multi-factorial and include disproportionate chronic disease prevalence, socioeconomic factors, as well as cultural and political influences. However, well-documented underlying factors are socioeconomic and discriminatory inequities at the individual, familial, and community levels (14–19).

**Figure 1:**
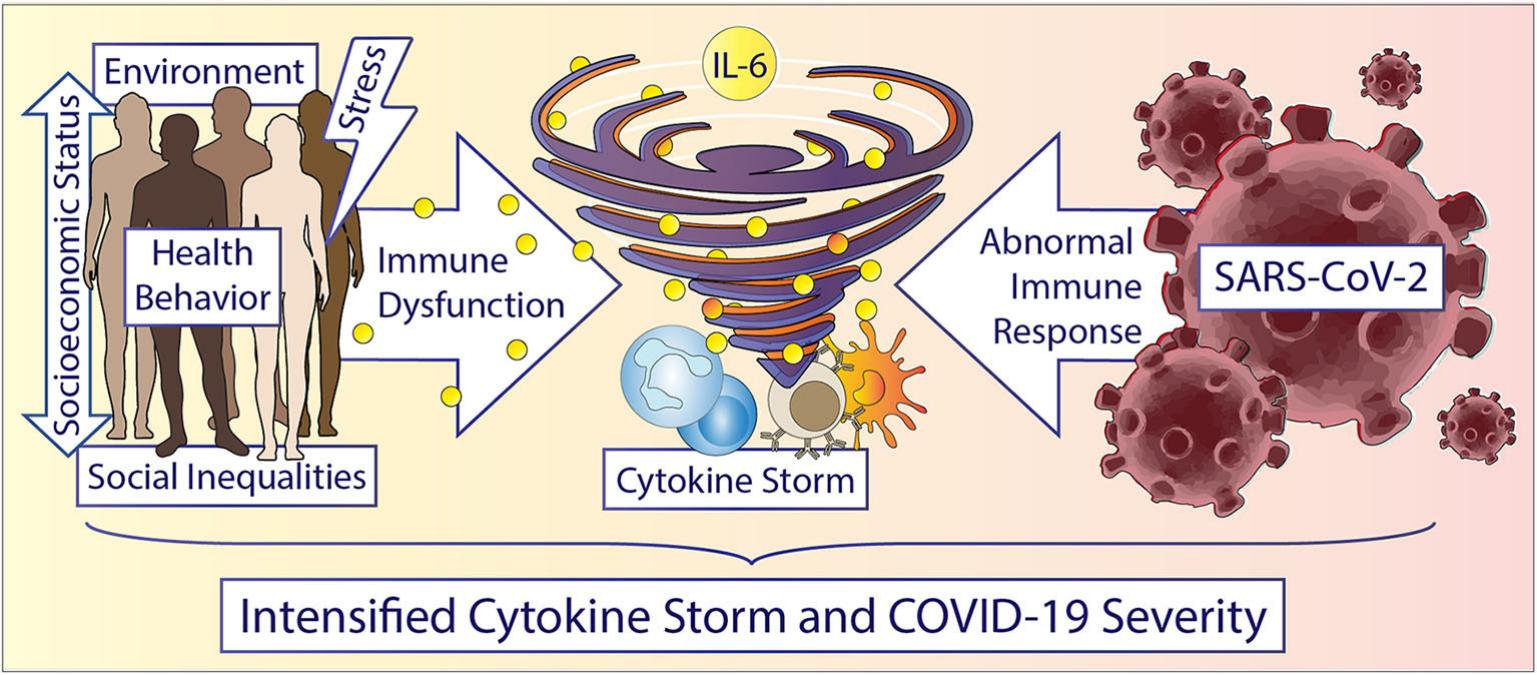
Accelerating the storm in vulnerable populations during COVID-19. Race/ethnicity, gender, and social determinants are important factors driving inflammation and immune cell dysfunction. Social determinants include but are not limited to: structural inequities, socioeconomic status, education, neighborhood environment, experiences of abuse, racism or other adversities, chronic stress, income, and education etc. Humans experiencing any of these social determinants can display increased inflammation, especially increased IL-6 levels and dysfunctional immune cells. Vulnerable patients potentially experience an accelerated cytokine storm during a COVID-19 infection due to a hyperactive and dysfunctional cytokine and immune response. Understanding the underlying cellular and immune related mechanisms in health disparities could potentially help in reducing disparate outcomes overall and in future pandemics.

Over the last 30 years, growing evidence in the literature has demonstrated that social determinants can induce a state of low-grade inflammation accompanied by a dysfunctional immune system in individuals experiencing adversities (20–22), but mechanistically, there is less work linking adverse psychosocial or environmental conditions (e.g., neighborhood deprivation, built and social environment) on a cellular and signaling pathway level. Additionally, very little is known about potential common dysregulated signaling pathways regarding immune cell dysfunction and inflammation between adverse psychosocial or neighborhood environmental conditions and other comorbidities known to affect COVID-19 severity. An understanding of the pathophysiology of COVID-19 requires an understanding of the interplay of SARS-CoV-2 virus with immune cells and inflammation which is slowly emerging in the literature (23). This understanding may guide therapeutics, translational studies, and multidisciplinary interventions that may mitigate some effects of COVID-19, while also elucidating mechanisms by which health disparities influence outcomes during pandemics and beyond for future targeted treatment. In this perspective, we present evidence of potential connections between the observed health disparities and COVID-19 progression/severity based on immune regulation and response, particularly from natural killer (NK) cells, monocyte/macrophages, and the cytokine, interleukin-6 (IL-6) (Figure 1). While we recognize that there are existing disparities in terms of SARS-CoV-2 susceptibility and detection, our intent with this perspective is to state that racial/ethnic health disparities in COVID-19 severity and mortality that are present in this pandemic may at least partially represent the biological consequences of social determinants of health, including socioeconomic and environmental inequities. Beyond pharmacologic therapies, behavioral interventions focused on reducing psychosocial stressors and accounting for existing social determinants in at-risk populations may help in mitigating the harmful immune and inflammatory responses.

## COVID-19 Related Cytokine Storm: A System out of Balance

The body's immune system is a well-controlled and balanced interplay of various players, involving cellular components (immune cells) and circulating proteins with pro- and anti-inflammatory function (cytokines and chemokines) (24). The immune system detects and neutralizes foreign pathogens, including viruses like SARS-CoV-2. Two major subsystems of the immune system exist in many species: the adaptive vs. innate immune system. The adaptive immune system is acquired and able to adjust with time, recognizing pathogens more specifically by developing a memory after the first encounter with a pathogen. In contrast, the innate immune system serves as the active defense against a foreign pathogen. Upon encountering a pathogen, immune cells are recruited to the site of infection due to chemokines released by the infected tissue resident cells. Immune cells are further activated to provide an adequate response by various pro-and anti-inflammatory cytokines also released by the infected tissue, regulating immune cell function as well as immune cell interplay, coordinating healing and clearance of the pathogen (25–27).

Immune cells include, but are not limited to, neutrophils, T cells, B cells, NK cells, and monocytes (25, 27, 28). Neutrophils are the most abundant immune cell in the human body and important in the early stages of inflammation. T cells, B cells, and NK cells are also called lymphocytes. T cells are divided into many different subclasses and are major cytokine producers, which is important for coordination of the overall immune response; T cells also exhibit killing abilities of cancerous or virus-infected cells. As a part of the adaptive immune system, B cells are antibody producing and secreting cells. NK cells exhibit different mechanisms of detecting virus-infected or cancerous cells as compared to T cells, subsequently killing cells detected as “abnormal,” and are generally divided into two subclasses: (a) the proliferative NK cells activating additional parts of the immune system by cytokine secretion, or (b) the cytotoxic NK cell which release cytotoxic granules that mediate NK cell killing. Monocytes are phagocytosing immune cells and are responsible for “cleaning” debris derived from NK cell- or T cell-mediated killing of infected cells. Monocytes can then present parts of the engulfed pathogens to T cells, further activating an immune response for clearing the infection. Monocytes can also exit the circulation and transmigrate into surrounding tissues, clearing debris and supporting healing of the infected and inflamed tissue. Furthermore, monocytes differentiate into macrophages mainly responsible for cleaning up debris by phagocytosis mechanisms. Additionally, monocytes are present in three different subtypes: classical, non-classical and intermediate, with each of them having distinct functions.

Cytokines are a small group of proteins secreted by cells to communicate with each other, regulating proliferation, differentiation, inflammatory responses, immune cell activation and recruitment, as well as angiogenesis (29, 30). Among cytokines within the human body, interleukin (IL)-6 is probably one of the more complex as it exhibits pro- and anti-inflammatory properties and plays an important role in vascular disease, pathologic lipid metabolism, insulin resistance, and several chronic lung diseases (31, 32), all known risk factors for COVID-19 morbidity and mortality (1). Regarding COVID-19, Herold et al. (33) reported in a recent publication that the risk for respiratory failure in confirmed COVID-19 patients is higher in patients with IL-6 levels above 80 pg/ml when compared to patients with lower IL-6 levels. Herold et al. suggested that IL-6 levels could be used as a biomarker for disease progression. An earlier study reported that the detectable serum viral load of COVID-19 was closely associated with drastically increased IL-6 levels in the critically ill (34). Activation of a coordinated cytokine response, especially in the case of an acute infection, is crucial in activating immune cells and, therefore, fighting the infection. During SARS-CoV-2 infections, a “cytokine storm,” or an overactive inflammatory response of the immune system (35), can occur (30, 35, 36). But important questions come to mind: How and why are some patients experiencing this cytokine storm and others don't? What is known from epidemiologic research that puts COVID-19 patients at risk for experiencing a cytokine storm and could this help understand existing COVID-19 severity and mortality disparities?

While genetics are certainly postulated to play an important role for the development of a cytokine storm, we would like to highlight in this perspective that social position, racial discrimination, adverse neighborhood environment conditions, or experiences of individual-level adversity could potentially increase the risk of COVID-19 progression and mortality by altering immune cell function and existing inflammation levels, therefore altering the crucial balance needed for a coordinated immune system response. Due to prior work of our laboratory, this perspective will focus on the potential role of monocytes and NK cells at the intersection of social determinants and COVID-19 outcomes. Additionally, we chose to focus on the role of IL-6 over other cytokines as (a) IL-6 is consistently shown in the literature to be altered by psychosocial stress or environmental factors, and (b) its importance in COVID-19 severity is highly suggested based on available treatments. Certainly, while this perspective focuses on monocytes, NK cells, and IL-6, it is important to note that other immune cells and cytokines or chemokines are also key players in the body's coordinated response to infection, are affected by adverse psychosocial or environmental conditions, and hence warrant acknowledgment and future investigation.

## Markers of Social Position and the Immune System

It is by now evident that racial/ethnic disparities in COVID-19 exist (4, 5, 14, 37) and there are indications that COVID-19 severity might differ depending on one's neighborhood deprivation and socioeconomic status (38–40). These studies highlight the importance to further understand the impact of social position on immune system function.

Amongst all immune cells, T cells, monocytes, and NK cells are taking a center stage in COVID-19 severity (41–44), recovery (45), and mortality (46), indicating that disturbed baseline T cell, monocyte or NK cell function might hinder adequate response to SARS-CoV-2 infection. In the past, our group and others have demonstrated racial/ethnic differences in immune cell distribution and cytokine levels (47–52). For instance, we and others showed shifts in monocyte subsets in non-Hispanic Blacks toward intermediate (CD14^−^/CD16^+^) and non-classical (CD14^+^/CD16^+^) monocytes accompanied by a decrease in classical monocytes (CD14^+^/CD16^−^) as compared to Caucasians. We hypothesize that this subset shift could relate to more severe COVID-19 outcomes in non-Hispanic Blacks, especially as the pro-inflammatory, non-classical (CD14^+^/CD16^+^) monocyte subset can play an important role in the inflammatory storm observed in severe COVID-19 (42) and has been postulated as potentially crucial in separating mild from severe COVID-19 cases (53). Additionally, we have recently demonstrated in a small hypothesis-generating study that the NK cell profile of non-Hispanic Black blood donors differs significantly from that of Caucasian blood donors (47). Overall NK cell proportions were increased in non-Hispanic Black blood donors. While increasing NK cell numbers might sound like an enhancement of immune cell function, the opposite is true when additional NK cell subset changes or functional changes are present. We found decreased levels of “cytotoxic” NK cells (CD56^hi^/CD16^dim^) and increased levels of “proliferative” NK cells (CD56^dim^/CD16^hi^) within the overall NK cell populations among non-Hispanic Black as compared to Caucasian blood donors (47). This shift in NK cell subsets is indicative of a loss of NK cell “killing” ability and could potentially be linked to a delayed or decreased response to an acute viral infection, like SARS-CoV-2.

A study published in Science in 2016 elegantly displayed how unequal social status with subsequent harassment during social interactions altered T-helper cells and NK cells (54); although this study was conducted in rhesus macaques and translation to humans has to be determined, these findings could potentially be of crucial importance for COVID-19 where T cells are particularly affected during the immune response in severe COVID-19 cases (42, 44, 55). Interestingly, socioeconomic differences in early life seem to drive epigenetic aging and DNA methylation of monocytes and therefore, alter monocyte functionality, potentially contributing to future health disparities (56, 57). In children with asthma, a risk factor for more severe COVID-19 disease, lower socioeconomic status (SES) was accompanied with worse quality of life and a shift of immune cell function toward more inflammatory monocytes and altered T-helper cell responses when compared to children of higher SES with asthma (58). Socioeconomic position across the life course also appears to be of crucial importance in shaping the immune cell landscape, with lower socioeconomic position across the life course being associated with greater baseline inflammation in adulthood (59). During COVID-19 progression, exaggerated baseline inflammation and a dysfunctional immune system could lead to an inadequate and, therefore, less effective immune response. This could partially explain increased COVID-19 severity and mortality in individuals experiencing disadvantages across the life course.

Leukocyte telomere length has been linked to immune cell dysfunction, various chronic diseases (60), and psychosocial stress [as summarized in (61)]; telomere length has been suggested as a new tool for immunoepigenetics (62). A recent study from our lab using data from the National Health and Nutrition Examination Survey demonstrated that living in socioeconomically disadvantaged neighborhoods associated with shorter leukocyte telomere length among U.S. adults, indicating the potential impact of adverse neighborhood conditions on immune cells (63). It is important to note that leukocyte telomere length has also been reported to have inconclusive results for some diseases (64) and certainly warrants future research as racial/ethnic differences have been shown when comparing telomere length and/or telomere shortening rate of Blacks to those of Whites (65–67). Overall, urgent studies are needed to investigate cellular signaling mechanisms by which markers of social position, including socioeconomic position and the neighborhood environment of individuals (68), shape the transcriptional identity of immune cells (69) to further understand the disparities in COVID-19 outcomes and potential targets for therapeutic intervention.

Racial/ethnic differences in IL-6 levels have been reported. IL-6 levels are higher in non-Hispanic Blacks when compared to Hispanics or non-Hispanic Whites after age adjustment (70). While this study did not adjust for gender, an impact of gender in IL-6 response to stressors has been reported (71), but evidence in larger studies is sparse. In another study, Blacks displayed higher IL-6 even after adjustment for socioeconomic factors (48). IL-6 is also the major cytokine associated with chronic psychosocial and environmental stress in epidemiologic studies. For example, the list of factors reported to increase circulating IL-6 levels include lower SES (72–74), higher levels of neighborhood deprivation, and lower levels of neighborhood safety (75). Additionally, elevated IL-6 levels are present in older adults living in communities with greater poverty and racial segregation (76). In another study, no baseline differences in circulating IL-6 levels in individual of lower or higher SES were found, but a prolonged and accelerated plasma IL-6 increase was observed when individuals of lower SES were subjected to an acute stressor (77). When Azad et al. analyzed the IL-6 release of stimulated PBMCs of children of various SES levels, they reported that PBMCs from those with lower SES displayed an increase of IL-6 release twice as high as PBMCs from children with higher SES, indicating excessive IL-6 production may be dependent on SES (78). These relationships between lower SES and increased inflammation in adulthood have been reported in other studies as well (59). Early childhood stressors can include exposure to poverty, war, and systemic racism, or lower education while an adulthood stressor could certainly include the COVID-19 pandemic. This indicates that COVID-19 patients who have previously experienced poverty, and/or institutional racism, especially at a young age (79), might react to COVID-19 with an overproduction of cytokines, including IL-6, accelerating the cytokine storm and worsening disease prognosis.

## Individual-Level Adversities and the Immune System

Individual-level markers of adversity include, amongst others, experiences of abuse, bereavement, relationship stress, limited social cohesion, loneliness, or sleep deprivation and have partially been linked to markers of social position and vice versa (80–84).

Epidemiologic data suggest relationships between the function of NK cell, monocyte/macrophage, and T cell populations and chronic psychosocial stress. For example, multiple psychosocial stressors like bereavement, relationship stress, depression, and generalized chronic mental stress are associated with a loss in NK cell function (85). A study focusing on loneliness found that NK cell numbers as determined by CD56/CD16 positive NK cells in flow cytometry decrease with increasing levels of loneliness (86). A potential source of confusion when examining the literature might be that markers of lower social status or adversity may correlate with higher numbers of overall immune cells. For example, the number of circulating NK cells overall has been demonstrated to increase in situations of acute mental stress or depression (87). In future studies, it will be important to expand the phenotypic characterization of immune cell subtypes and to measure actual immune cell function, as a simple increase of a particular immune cell can still be accompanied by a decrease in important subsets of cells with a required specific function (i.e., attacking a virally infected cell).

When PBMCs were isolated from middle-aged women with various levels of physical assault history, *in vitro* production of IL-6 upon stimulation was significantly associated with the level of assault experienced (88). Similar results were observed for individuals who have experienced stalking (88), indicating a dysfunctional immune system in women experiencing assault. Sleep deprivation as well as loneliness (89) have also been demonstrated to alter immune cell IL-6 production, further strengthening the evidence that individual-level adversity alters immune cell function and hence could predispose an individual to greater risk for increasing COVID-19 severity (90).

Social cohesion is another factor of importance particularly in times of social distancing. Social cohesion has been shown to be accompanied by decreasing IL-6 levels, especially amongst African American women (91). Additionally, increasing social support in parents of children with cancer has been demonstrated to attenuate glucocorticoid resistance and subsequent IL-6 production, while no impact of social support could be found regarding TNFα or IL-1β (92). Furthermore, hopelessness in adolescent girls in the setting of bullying was also shown to be associated with increased IL-6 levels (93). During a pandemic, it is likely that anxiety levels, hopelessness, and psychosocial distress in individuals are increasing. Anxiety is a psychosocial factor shown to associate with increased IL-6 levels (94) independent of depressive symptoms (95), potentially further accelerating COVID-19 severity.

Interestingly, it has also been shown that increased IL-6 levels can be found in night shift workers when compared to day shift workers, while CRP, a general marker of inflammation often measured in the clinical setting did not reveal significant differences (96). Higher IL-6 levels were also detected with sleep deprivation and fatigue (97), bullying (93), or sexual abuse (98). In non-Hispanic Black women, higher IL-6 levels were found in those anticipating racism threats (99), with perceived lifetime discrimination (100), or experiencing ongoing racial discrimination (101). Intriguingly, there is growing evidence that stress in childhood accelerates IL-6 production when exposed to an acute stressor in adulthood, indicating that childhood stress may manifest in adults as accelerated IL-6 responses to acute stressors (102).

These findings highlight the importance of analyzing a wide variety of cytokines to determine the impact of “stressors” on health and in health disparities, and to potentially identify a few cytokines which could be useful as biomarkers in clinical practice estimating an individual's risk for adverse health outcomes.

## Behavioral Interventions Impacting IL-6 Levels and the Immune System

Several interventions have been shown to decrease IL-6 levels; in particular, behavioral interventions around physical activity, diet, and behavioral approaches to stress could be of benefit by potentially dampening the IL-6 production and other cytokine changes in COVID-19 progression. Dietary behaviors and physical activity, in particular, may serve to provide a two-way approach to combat health disparities during the COVID-19 pandemic, principally through their ability to prevent underlying chronic diseases but also through potential protective changes in the immune and inflammatory responses. In older adults, increasing exercise levels long term has been beneficial in decreasing IL-6 levels (103), a finding supported by other studies (104), which highlights the importance of long term regular exercise, even in times of a pandemic. In a study focusing on acute psychosocial stress, increased self-compassion was also accompanied by a decrease in the IL-6 response (105). Another study explored monocyte subsets and inflammatory markers in connection with healthy diet habits. The authors found that adherence to nutritional recommendations resulted in lower levels of inflammatory markers and non-classical monocytes. Moreover, a study by Mayr et al. demonstrated that altering diet by reducing the dietary inflammatory index correlated with a significant reduction in serum IL-6 levels after 6 months of dietary change (106). Several interventions have also been shown to alter NK cells; for instance, daily nature walks have been shown to increase NK cell numbers and functionality (107) and support for psychosocial well-being increases NK cell cytotoxicity (108). As mentioned above, social cohesion is a critical component affecting the immune system, indicating the potential importance of online or mobile health support groups even in times of necessary social distancing.

Exploring the role of behavioral interventions may also provide insight into the connection between the immune system, inflammation and the contextual environmental and extrinsic factors that underlie health disparities. For example, the presence of low-grade inflammation in young adults experiencing racism was shown to be less extensive if a positive racial identity was present (79).

Diabetes itself is linked to higher circulating IL-6 levels, and in turn, diet has been linked to IL-6 as well. The connection between the two seems obvious and could potentially be a part of the reason why certain diseases in vulnerable populations lead to worse COVID-19 outcomes. Healthy food choices are also influenced by multiple parameters like socioeconomic status or one's neighborhood environment (109), with most predominantly Black urban neighborhoods having limited fresh food access. As an example of the potential confluence of stress, food access, and racism affecting health behavior that influences chronic disease, non-Hispanic Black women with diabetes reported that experienced racism led to maladaptive coping strategies which included unhealthy food choices and that these choices affected diabetes self-management (91, 110). Lastly, interventions should certainly incorporate the element of hope, as hope, optimism, and happiness are essential components to human health and have been shown to be accompanied by decreasing IL-6 levels (111, 112). This might seem like a list of unbreakable, vicious cycles pre-dispositioning vulnerable populations to COVID-19. However, this is not the intention of this perspective article. Especially in times of social distancing, small behavioral interventions to promote healthy diet, physical activity, or reduce stress might make a difference in COVID-19 outcomes.

## Conclusion

In conclusion, we must consider how we can address social determinants of health that promote psychosocial stress and disparate outcomes regarding disease severity and mortality in COVID-19. There is clear evidence that social disadvantages and stressors experienced in early life will shape the immune cell function and cytokine levels across the life course. The COVID-19 pandemic highlights several important questions to consider. Could anti-inflammatory treatments, especially clinical trials targeting IL-6, the IL-6 receptor, or the IL-6 tyrosine kinase signaling pathway, be of potential benefit especially for the most vulnerable in our society? How can we guarantee clinical trial access to minority-serving institutions and clinical providers around the country to ensure that study participants of all races/ethnicities and socioeconomic backgrounds are enrolled in these critical COVID-19 related clinical trials? Can we phenotype immune cells and/or determine cytokine levels in bio-banked samples from existing clinical trials or health care system cohorts and combine these data with electronic health record data or publicly accessible data to evaluate the impact of social determinants on underlying signaling pathways potentially accelerating poor COVID-19 outcomes? Can we leverage existing epidemiological cohort studies or behavioral interventions to identify participants that have been affected by COVID-19 and investigate factors contributing to COVID-19 health disparities by analyzing participants' disease progression and outcomes in relation to psychosocial stressors, neighborhood environment, health behaviors, chronic diseases or inflammatory markers? Some of this work has already begun with epidemiologic cohorts like the Multiethnic Study of Atherosclerosis and the Nurses' Health Study.

Looking ahead to the months and years to come, we need to consider more interdisciplinary approaches to evaluate cellular signaling pathways involved in disparate outcomes in COVID-19 that may serve as intervention targets. This work may also elucidate important targets for reducing racial/ethnic health disparities in chronic health conditions. Future clinical trials need to incorporate rigorous qualitative methodologies and validated quantitative questionnaires regarding study participants' background like socio-demographics, address data to link with data on neighborhood traits, and questions about adverse early childhood and life experiences, including psychosocial stressors. Behavioral interventions should be designed to gather phenotypic data and understand cellular pathways that may be most impacted by changes in health behavior. Evaluation of these data could add tremendously to understanding the contribution of social determinants of health to clinical outcomes and how their contribution might be attenuated by intervention. Certainly, it would also be desirable if epidemiological studies and behavioral interventions could incorporate a more detailed translational research component by detecting cytokine levels, immune cell phenotypes, immune cell function, and the underlying cellular signaling pathways, to potentially identify common regulators between diseases allowing for better development of treatment strategies or interventional therapies. While several epidemiologic studies have been conducted over the last 30 years that include translational research, it will be important to incorporate deeper immune cell phenotyping, characterization of immune cell functionality, and larger cytokine profiling across epidemiologic studies for data harmonization that allow for inter-study comparison. For basic research, it will be particularly important to address basic research questions regarding the impact of stress and stressors on cellular function especially in the most vulnerable patients affected by health disparities. A more intense focus should involve psychoneuroimmunology (113–115) to further identify underlying mechanisms and signaling pathways. As none of us can be an expert in every field, interdisciplinary collaborations are of crucial importance to make this kind of in-depth, overarching research feasible and to potentially better understand the underlying mechanisms for health disparities.

In this perspective we have highlighted inflammation and immune cell changes as potential mediators of the relationships between exposure to adverse social conditions and COVID-19 severity and outcomes. It is known that existing inequities, may lead to the disproportionate burden of chronic diseases, such as asthma (116–118), Type 2 diabetes (119, 120), obesity (121–126), and cardiovascular disease (127–129). In turn, these chronic diseases could mediate associations between social factors and worsening COVID-19 severity and outcomes (1, 130). However, while we believe that comorbidities are of great importance in understanding COVID-19 progression, formal mediation analyses in epidemiologic studies can begin to determine whether biomarkers of immune system alterations and inflammation may mediate associations between social conditions and COVID-19 severity and outcomes, independent of comorbid conditions. This epidemiologic work could provide direction to translational research to further understand the underlying mechanisms by which immune cell changes and inflammation might be altered by social determinants.

In the future, it will be important for countries to make data regarding racial/ethnic disparities available and accessible to the scientific community to allow further understanding of the impact of social determinants of health on COVID-19 progression, severity, and mortality. Furthermore, this type of data could be utilized to determine the impact of existing health care systems, access to healthcare, testing and treatment, as well as population structure on health disparities, ultimately allowing for improved future strategic planning in pandemics.

The need to work together as a world team of science is now more crucial than ever to understand COVID-19 progression in the most vulnerable, giving hope for the future of patient care and treatment development. Hope is essential to make it through this pandemic and its tragic consequences and for us to build a world where future pandemics are not only less likely but less disparate in their impact.

## Data Availability Statement

The original contributions presented in the study are included in the article/supplementary material, further inquiries can be directed to the corresponding author/s.

## Author Contributions

YB and TP-W conceptualized and wrote the article. NF and GW critically reviewed and revised the article. TP reviewed the article and developed the graphical abstract. All authors read the article and agreed to its submission.

## Conflict of Interest

The authors declare that the research was conducted in the absence of any commercial or financial relationships that could be construed as a potential conflict of interest.
